# From Citizenship Pressure to Pro-Group Unethical Behavior: The Dual-Stage Moderating Role of Self-Serving Political Will

**DOI:** 10.3390/bs13070544

**Published:** 2023-06-29

**Authors:** Hantai Zhang, Minqiao Hu, Xin Liu, Xuan Yu, Jinyu Xie

**Affiliations:** 1 Business School, Sichuan University, Chengdu 610064, China; 2 School of Economics and Management, Southwest Petroleum University, Chengdu 610500, China

**Keywords:** citizenship pressure, political will, moral disengagement, pro-group unethical behavior

## Abstract

Drawing upon social cognitive theory, our study proposes a dual-stage moderated mediation model that utilizes moral disengagement as the mediator and self-serving political will as the moderator to investigate whether, how, and when team-oriented citizenship pressure leads to pro-group unethical behavior. Data were collected through questionnaires from 527 Chinese employees in various industries. Amos and Process macro were used to test the model’s fit and hypotheses, respectively. The results showed that citizenship pressure has a positive effect on pro-group unethical behavior through moral disengagement. Moreover, self-serving political will is a positive moderator in both the first and second stages, as well as in the mediation effect. This study extends the related research field by linking citizenship pressure and political will with moral disengagement and pro-social unethical behavior, responding to some academic calls. When faced with team-oriented citizenship pressure, team members with high self-serving political will may become a sharp edge that stabs at other competing teams. Managers at the team and organizational levels can intervene in different ways depending on their constructive or destructive management goals.

## 1. Introduction

Both academia and industry acknowledge that in increasingly competitive environments, managers tend to encourage employees to contribute beyond their formal job duties to achieve higher collective performance at lower costs [1,2]. Scholars have defined this pressure employees feel to engage in organizational citizenship behaviors (OCBs) to meet job requirements as citizenship pressure, and have revealed that while citizenship pressure can guide employees to engage in more OCBs, it can also lead to adverse consequences such as work–family conflict, job stress, turnover intention, and facades of conformity [3,4,5].

This study further investigates the dark side of citizenship pressure, and examines a new potential result that undermines organizational stability from a team perspective: team members’ pro-group unethical behavior. Pro-group unethical behavior refers to behaviors that violate moral, legal, or behavioral codes in an attempt to contribute to the well-being of their group [6]. Numerous studies have found that employees may use unethical behaviors to cope with challenges in the workplace [7,8,9,10]. In the practice of team competition, when employees, driven by citizenship pressure from their workgroup, are committed to role-exceeding behaviors that benefit team performance, they may also choose unconventional and unethical means under certain conditions, such as attacking other teams to gain a performance advantage for their own team. Considering that pro-social unethical behavior in the workplace may impose costs on the target beneficiary group [11], and pro-group unethical behavior implies internal consumption of the organization, both team and organization level managers need to understand whether, how, and when citizenship pressure may lead to pro-group unethical behavior. However, this specific phenomenon does not seem to have been discussed, although existing studies have focused on the impact of workplace stressors such as performance pressure and stretch goals on employee unethical behavior [8,9,10].

To address this gap, our study draws on social cognitive theory and constructs a two-stage moderated mediation model to test the relationship and explore its mechanisms and boundary conditions. Social cognitive theory summarizes the techniques of moral disengagement and emphasizes that moral behavior is a product of the interaction between individuals and social influences [12,13]. We hypothesize that citizenship pressure not only guides members to contribute to the team but also gives them good reasons to disengage from their moral responsibility, leading to a stronger tendency toward moral disengagement, thereby making them feel justified in engaging in pro-group unethical behavior. Specifically, citizenship pressure from the team, with its features of team-oriented contribution, external pressure, and extra-duty requirement, enables members to use moral justification, displacement of responsibility, dehumanization, and attribution of blame techniques to escape moral responsibility and engage in pro-group unethical behavior. In other words, moral disengagement serves as a mediator. Additionally, against the background of team-oriented citizenship pressure as a social influence, we believe that self-serving political will as a motivation characteristic is a relevant individual factor. Pro-group unethical behavior is a social influence action that has both risks and benefits. Self-serving political will, as a critical factor that affects individual social influence opportunity assessment and action decision-making [14], may play a moderating role in the process of citizenship pressure triggering pro-group unethical behavior. In particular, the self-interested motivation, need to achieve, and risk-taking propensity of employees with high self-serving political will make them more likely and more inclined to set aside moral self-regulation and take more risks to implement pro-group unethical behavior. That is, self-serving political will is a positive moderator in both stages. Furthermore, we examined its moderating role in the mediating effect.

This study has theoretical and practical contributions. First, we depicted team-oriented citizenship pressure and revealed the positive predictive effect of citizenship pressure on pro-group unethical behavior and the mediating role of moral disengagement. These findings extend our understanding of the consequences of citizenship pressure and the antecedents of pro-social unethical behavior, and future research can explore more inherent relationships. Second, by using self-serving political will as a moderator in two stages, this study links political will with moral disengagement and pro-social unethical behavior. This forms our response to the call to explore how individual differences adapt to the self-regulation model of morality [15], and to identify the self-serving motivation for pro-social unethical behavior [16]. Third, this study discusses the opportunities and challenges that team members with high self-serving political will with citizenship pressure bring to teams and organizations. For team managers, subordinates with high self-serving political will combined with team-oriented citizenship pressure can become a sharp tool for teams to use when competing with other teams. Therefore, managers at different levels can adjust their management methods according to their different management goals of construction or destruction.

## 2. Theoretical Background and Hypotheses

### 2.1. Citizenship Pressure and Pro-Group Unethical Behavior

Although citizenship pressure can stimulate employees to engage in more OCBs, it may also lead to many adverse outcomes, such as work–family conflict, job stress, turnover intention, and facades of conformity [3,4,5]. These consequences are related to personal costs, which are determined by the concept’s connotation. Citizenship pressure may make employees realize that performing formally defined job duties is not enough to earn the reputation of a valuable team member, and that they may have to consume personal resources to make more contributions outside their formal role in order to meet organizational expectations [3,17].

However, contributions can be achieved not only through regular OCBs, but also through unconventional pro-social unethical behavior. Pro-social unethical behavior is defined as work behavior that aims to benefit others but violates social core values, norms, laws, or appropriate behavioral standards [18]. Depending on the focus of attention, it can be divided into pro-organization unethical behavior, pro-group unethical behavior, pro-leader unethical behavior, etc. [16]. The employee’s original intention when engaging in pro-social unethical behavior is for the benefit of others, but sometimes the target beneficiary group may lose more than it stands to gain [11]. Therefore, the academic community has focused on exploring the influencing factors of this type of behavior, and has identified antecedents such as organizational identification, supervisors’ pro-social unethical behavior, and high-performance work systems [19,20,21].

Social cognitive theory recognizes the status of the social environment in influencing individual behavior [22]. This study believes that citizenship pressure, as a social environment, is also a potential antecedent of pro-social unethical behavior, and it is understandable that employees influenced by citizenship pressure may implement pro-social unethical behavior as a means of contributing. On the one hand, conceptually, the “citizenship” part of citizenship pressure is inherently linked to the “pro-social” part of pro-social unethical behavior. Citizenship pressure requires employees to engage in more OCBs and perform some voluntary beneficial behaviors for the collective [3], which is consistent with the connotation of pro-social unethical behavior, that is, taking action for the wellbeing of others [18]. On the other hand, according to the challenge–barrier stressor framework, citizenship pressure is a challenging stressor with clear and actionable coping strategies (OCBs), and individuals tend to face this work pressure through performance-enhancing efforts rather than direct withdrawal strategies [23,24]. At the same time, a large body of research suggests that employees may engage in unethical behavior to cope with workplace challenges such as performance pressure and stretch goals [7,8,9,10], and employees who invest in unethical behavior may indeed receive better performance evaluations [25].

In the context of team environments, citizenship pressure coming from a team is more likely to lead to pro-group unethical behavior. According to social influence theory, the degree of social influence is positively related to its immediacy (or spatiotemporal proximity) to the target of influence [26]. From this perspective, it can be argued that most of the social influence that employees receive in their work environment comes from team leaders and colleagues, including citizenship pressure. In practice, team leaders often emphasize that employees should contribute more to the team’s performance when implementing team goals (which may not be fully aligned with organizational goals). As such, when citizenship pressure comes mainly from team goals or team leaders and colleagues, rather than organizational goals or the entire organization, immoral behavior that is oriented towards making extra contributions may extend from pro-organizational behavior to pro-team behavior. In other words, team members may be more willing to engage in unethical behavior that benefits their team rather than their organization when the source of citizenship pressure is the team.

**Hypothesis 1 (H1).**  *Citizenship pressure has a positive effect on pro-group unethical behavior.*

### 2.2. Moral Disengagement as a Mediator

Bandura [22] proposed the concept of moral disengagement under social cognitive theory, which depicts the process by which people weaken the moral self-control mechanisms that can prevent them from engaging in unethical behavior in various ways. By escaping the self-condemning reactions that arise from violating their moral standards, people can engage in unethical behavior with a clear conscience. Specifically, these disengagement techniques include linking harmful behavior with valued goals to transform harmful behavior into moral behavior, deflecting and transferring responsibility to hide one’s role as an aggressor, distorting or ignoring harmful effects on others, and dehumanizing and blaming the victim through accusations and depersonalization [22]. The moral disengagement mechanism of social cognitive theory is an influential way of explaining unethical behavior [15], and an effective way of explaining pro-social unethical behavior induced by stress [16]. For example, Chen and Chen [8] pointed out that employees’ performance pressure may positively predict their pro-organizational unethical behavior through moral justification (e.g., “I am doing this for the benefit of the organization, not myself”).

Similarly, starting from the concept itself [3], we speculate that citizenship pressure provides members with good reasons to evade moral responsibility, and facilitates their moral disengagement tendencies in three aspects. Firstly, the feature of team-oriented contributions within citizenship pressure activates members’ moral justification techniques. Citizenship pressure requires members to make extra contributions to the team’s wellbeing, and members can moralize unethical behavior by emphasizing the value of their actions to the team. For example, “I am doing this for the benefit of the team, not for my own benefit.” Secondly, the feature of external pressure activates members’ displacement of responsibility techniques. Because citizenship pressure comes from the leader’s excessive work demands and colleagues’ excessive dedication, members can attribute the use of unethical means to others. For example, “I am doing this because my leader always demands that I make more contributions to the team, and I have no choice.” Thirdly, in the context of team competition, the feature of extra-duty requirements activates members’ dehumanization and attribution of blame techniques. If other teams’ interest conflict with those of one’s own team, members may perceive citizenship pressure as originating from the competitive team. As a result, members not only view members of the competing team as outsiders, but also as enemies, believing that they deserve to be attacked. For example, “Anyway, they are competitors” or “I am under such great pressure, which is entirely due to their competition with us.” Acknowledging moral disengagement’s significant predictive role in unethical behavior [15], we propose that

**Hypothesis 2 (H2).**  *Moral disengagement mediates the relationship between citizenship pressure and pro-group unethical behavior.*

### 2.3. Self-Serving Political Will as a Moderator

Studies examining the effects of personal and situational factors on moral phenomena would more directly capture the interactive nature of our moral selves in social cognitive theory [13,15,27]. Given that pro-group unethical behavior is a social influence action that combines risks and benefits, and that political will is a key factor influencing an individual’s evaluation of social influence opportunities and decision-making [14], political will may play a moderating role in the process of triggering pro-group unethical behavior in the context of citizenship pressure. Treadway [28] defined political will as the motivation for strategic, goal-oriented behavior that promotes an individual’s personal agenda and goals, which essentially involve risks associated with relationship or reputational capital. Political will is an important individual difference in employees’ survival and development in organizational politics [29,30], and is a prerequisite for implementing impression management, political behavior, and more general social influence actions [14,31,32].

It is worth noting that quantitative research on political will has been constrained by the lack of standardized scales until Kapoutsis et al. [33] created a political will scale with both self-serving and benevolent aspects. Self-serving political will and benevolent political will, which represent self-centered and other-centered motives, respectively, differ in terms of relevant individual characteristics, influence strategy preferences, and job outcomes [33], and it is necessary to distinguish between them. Regarding our study, according to published evidence, self-serving political will is a more appropriate boundary condition. First, a meta-analysis by Kish-Gephart et al. [34] showed that Machiavellianism is positively correlated with various unethical choices, and only self-serving political will, not benevolent political will, is positively correlated with Machiavellianism on the two dimensions of political will [33]. At the same time, when employees have opportunities for self-interest, their motives for pursuing social influence and personal performance may lead to unethical behavior [35], and self-serving political will, not benevolent political will, is positively correlated with the need for power and achievement [33]. Additionally, benevolent political will predicts more influence strategies that are beneficial to the target (e.g., exchanging benefits), while self-serving political will mainly predicts coercive influence strategies for personal benefit (e.g., asserting one’s stance) [33]. Even for the overall performance of the team, behaviors that discredit and exclude other teams are not benevolent.

Self-serving political will makes it easier and more necessary for team members under citizenship pressure to shirk their moral responsibility. On the one hand, the self-interested motivation feature of self-serving political will implies a more easily discarded ethical standard. Moore, Detert, Trevino, Baker, and Mayer’s [15] analysis suggests that Machiavellianism can significantly predict moral disengagement because Machiavellians have different moral standards from normally socialized individuals, and have internalized pursuing self-interest as a new moral standard. In this case, the moral disengagement mechanism may be easily used to overthrow the moral self-regulation brought about by social moral standards. Similarly, political will can be reflected in an individual’s functional, ethical, and emotional responses to political behavior [36]. Strong political will means that individuals are more ethically tolerant of self-interested behavior that sacrifices the interests of others, and are more likely to be exempt from moral condemnation and self-sanctioning. As an acquired individual difference [32], the process of political will development may also be a process of lowering moral standards, which in turn will enhance the tendency toward moral disengagement. On the other hand, the need to achieve feature of self-serving political will makes members more inclined to shirk moral responsibility. Members with higher self-serving political will have a stronger need to achieve [33]. When encouraged to make extra contributions to the team, they are more likely to desire given outcomes, and need to temporarily set aside moral standards. Therefore, this study proposes:

**Hypothesis 3 (H3).**  *Self-serving political will positively moderates the relationship between citizenship pressure and moral disengagement. A member with higher self-serving political will is more likely to morally disengage under citizenship pressure, and one with lower will is less likely.*

At the same time, self-serving political will makes members with moral disengagement tendencies more daring when participating in pro-group unethical behavior. On the one hand, political will can predict the occurrence of unethical actions. Scholars have recognized the correlation between political behavior and unethical outcomes [31], as well as the intervention of moral levels in political behavior [37]. Doldor [38] pointed out that accepting the ethical risks of political actions is a required course for leaders, from the politically naive to the politically mature. According to Mayes and Allen’s [39] view, political behavior is an individual’s management of influence to achieve goals that are unrecognized or to achieve recognized goals through unrecognized means of influence. Meanwhile, pro-group unethical behavior (e.g., discrediting and excluding other teams) can essentially belong to the broad category of social influence action [14]. Pro-group unethical behavior implies organizational infighting, and is often not recognized by the organization. From this perspective, pro-group unethical behavior and political behavior have similarities, in that they can both be called unethical social influence actions. Therefore, political will as a typical driving factor of political behavior may also stimulate more pro-group unethical behavior. On the other hand, the risk preference feature of self-serving political will makes members more daring when participating in pro-group unethical behavior. According to the moral view of social cognitive theory, an individual’s unethical behavior is a function of self-sanctioning and social sanctions [12]. Social sanctions partially reflect the social riskiness of unethical behavior, and self-serving political will is negatively correlated with risk aversion [33]. Compared with individuals with low self-serving political will, those with high self-serving political will, after developing a tendency for moral disengagement, will be more willing to take risks of unethical and unconventional means in order to achieve team goals.

**Hypothesis 4 (H4).**  *Self-serving political will positively moderates the relationship between moral disengagement and pro-group unethical behavior. A member with higher self-serving political will is more likely to participate in pro-group unethical behavior after moral disengagement, and one with lower will is less likely.*

Furthermore, by combining H2, H3, and H4, a moderated mediated effect can be inferred. The self-interested motivation, achievement need, and risk-taking propensity features of team members with high self-serving political will make the process of using citizenship pressure to shift moral responsibility for pro-group unethical behavior smoother, as they are more likely, more inclined, and will dare to use unethical means that harm other teams to serve the interests of their team. In contrast, team members with low self-serving political will have difficulty accepting risky and self-serving social impact behaviors on ethical and emotional grounds [36]. They are unlikely to consider aggressive contributions through shifting moral responsibility, even under demanding work requirements that emphasize dedication.

**Hypothesis 5 (H5).**  *A member’s self-serving political will positively moderates the mediating role of moral disengagement between citizenship pressure and pro-group unethical behavior. When a member’s self-serving political will is higher, the mediating effect is stronger, and when lower, is weaker.*

**Figure 1:**
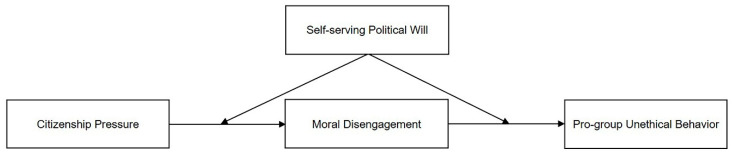
Conceptual model.

In summary, the conceptual model of this study is shown in Figure 1.

## 3. Methods

### 3.1. Sample and Procedure

The research team was invited to conduct consulting work, and the survey was mainly conducted through the convenience of the consulting project. The entire data collection process lasted about three months, with 28 organizations from multiple Chinese cities (such as Shanghai, Chengdu, and Chongqing) and multiple industries (such as new energy, manufacturing, and information technology) participating in the survey. Prior to data collection, the research team communicated with the organization’s contact person about the survey design, and obtained informed consent. In addition to the instructions included in the questionnaire, the research team provided on-site training and answered questions for participants to ensure that they understood the questionnaire correctly. At the same time, to ensure that participants could express their true state without any concern, the seats were set with a safe distance, and the questionnaire was collected directly by the research team. In addition, the data were kept strictly confidential, and only used for academic purposes. Participants were asked to report personal information and rate team-oriented citizenship pressure, moral disengagement, pro-group unethical behavior, and self-serving political will.

After the collection work was completed, problematic questionnaires (such as incomplete or overly regular questionnaires) were further excluded. Finally, 527 questionnaires out of 681 were used as the research sample, with an effective response rate of 77.386%. Among these 527 participants, 37.6% were male, with an average age of 30.59 years, and the majority (60.8%) of participants had achieved a bachelor’s degree (or above), with an average tenure of 6.69 years.

### 3.2. Measures

A five-point Likert scale was used for all variables except demographic variables, ranging from 1 (strongly disagree) to 5 (strongly agree). All scales were originally developed in English, and a back-to-back translation procedure was used to ensure the equivalence of measures between the English and Chinese versions of the survey instrument [40].

#### 3.2.1. Citizenship Pressure

Citizenship pressure was measured using an eight-item scale adapted from Bolino, Hsiung, Harvey, and LePine [17]. Respondents were asked to fill out this scale based on their perceptions of the team. One example item is “I feel a lot of pressure to go the extra mile by doing a lot of things that, technically, I do not have to do”. The Cronbach’s alpha for this scale was 0.915.

#### 3.2.2. Self-Serving Political Will

Kapoutsis, Papalexandris, Treadway, and Bentley [33]’s original four-item scale for self-serving political will was adopted. One example item is “Prevailing in the political arena at work would prove my competence”. The Cronbach’s alpha for this scale was 0.958.

#### 3.2.3. Moral Disengagement

We measured moral disengagement using the eight-item scale created by Moore, Detert, Trevino, Baker, and Mayer [15]. One example item is “People should not be held accountable for doing questionable things when they were just doing what an authority figure told them to do”. The Cronbach’s alpha for this scale was 0.958.

#### 3.2.4. Pro-Group Unethical Behavior

We adapted the five-item pro-group unethical behavior scale developed by Thau, Derfler-Rozin, Pitesa, Mitchell, and Pillutla [6]. One example item is “Bad-mouthing another team or another team member to take them out of the competition for opportunities in the company”. The Cronbach’s alpha for this scale was 0.964.

#### 3.2.5. Control Variables

Demographics (gender, age, education, and tenure) were included as control variables. Prior studies have suggested that these factors may affect unethical behaviors [34]. Gender was measured as a dichotomous variable, coded as 1 for men and 2 for women. Education was divided into five categories, ranging from high school to junior college, college, bachelor’s, master’s, and doctorate, labeled 1–5.

## 4. Results

### 4.1. Confirmatory Factor Analysis

To assess the discriminant validity of the measurement variables, we used a series of confirmatory factor analyses through IBM Amos v. 24.0, and the results are listed in Table 1. The baseline model, a four-factor model, presents a fair model fit (χ^2^ = 849.895, df = 269, RMSEA = 0.064, CFI = 0.956, IFI = 0.956, TLI = 0.951) while the three-factor, two-factor, and one-factor models fit badly.

### 4.2. Common Method Variance

Since the data were collected through a cross-sectional, self-reported survey, there might be a common method variance problem. Thus, following the suggestion of Podsakoff et al. [41], we added a latent common method factor into the hypothesized four-factor model on IBM Amos v. 24.0. This model fits the data slightly better (χ^2^ = 630.371, df = 244, RMSEA = 0.055, CFI = 0.971, IFI = 0.971, TLI = 0.964) than the four-factor model does. Nevertheless, the change in all fitting indexes was less than 0.02. Hence, common method variance was not a serious problem in the testing of our hypotheses.

### 4.3. Descriptive Statistics Analysis

The mean, standard deviation, correlation, and reliability of the variables are shown in Table 2.

### 4.4. Hypotheses Testing

We estimated two models, Model 4 and 58, based on Process macro v. 4.0, developed by Hayes [42]. The results are shown in Table 3.

The total effect of citizenship pressure on pro-group unethical behavior (β = 0.369, *p* < 0.001) supported H1. Citizenship pressure was positively related to MD (β = 0.341, *p* < 0.001), and moral disengagement was positively related to pro-group unethical behavior (β = 0.751, *p* < 0.001). An additional bootstrapping procedure revealed that moral disengagement mediated the relationships between citizenship pressure and pro-group unethical behavior (indirect effect = 0.256, 95% BC CI = [0.175, 0.346]). Thus, H2 was supported.

In support of H3, there was a statistically significant interaction between citizenship pressure and self-serving political will on moral disengagement (β = 0.078, *p* < 0.05). The simple slope tests show that the effect of citizenship pressure on moral disengagement was significant for members who reported high self-serving political will (simple slope = 0.285, *p* < 0.001) and non-significant for those who reported low self-serving political will (simple slope = 0.085, *p* > 0.10). These results supported H3.

**Figure 2:**
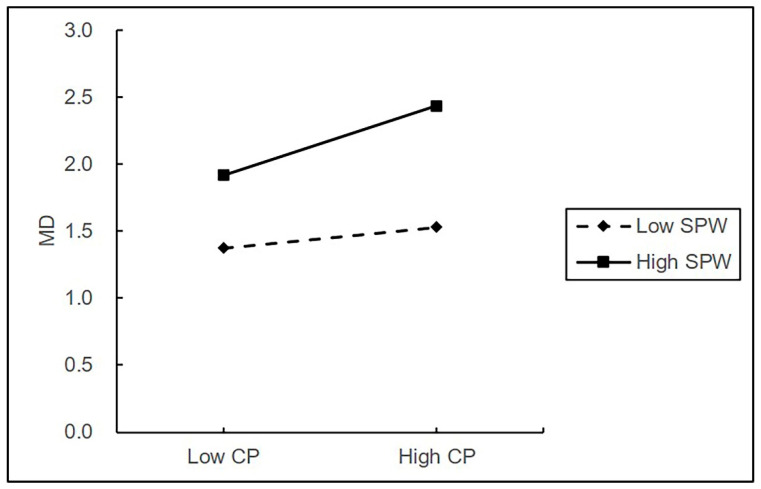
The moderating effect of self-serving political will on the relationship between citizenship pressure and moral disengagement.

**Figure 3:**
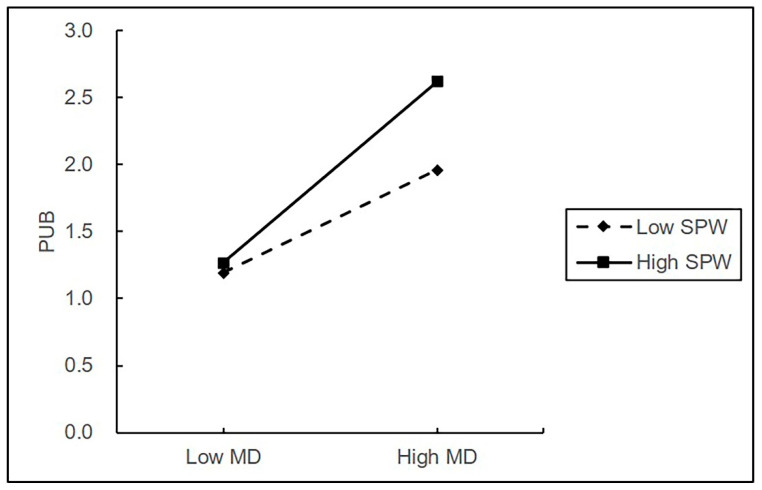
The moderating effect of self-serving political will on the relationship between moral disengagement and pro-group unethical behavior.

In support of H4, there was a statistically significant interaction between moral disengagement and self-serving political will on pro-group unethical behavior (β = 0.121, *p* < 0.001). Confirmed with simple slopes, there was a stronger effect of moral disengagement on pro-group unethical behavior (simple slope = 0.723, *p* < 0.001) at high compared to low levels of self-serving political will for pro-group unethical behavior (simple slope = 0.411, *p* < 0.001), supporting H4. As shown in Figure 2 and Figure 3, the relationships between citizenship pressure, moral disengagement, and pro-group unethical behavior were plotted at high and low values of self-serving political will, defined as one standard deviation above and below the mean value, respectively.

Finally, the hypothesized dual-stage moderated mediation effect was examined. In support of H5, the positive indirect effect of citizenship pressure on pro-group unethical behavior through moral disengagement (β = 0.105, 95% BC CI = [0.057, 0.160]) was found to be significantly stronger when self-serving political will was higher at both the first stage and second stage of the model. The difference between when self-serving political will is high at both the first stage and second stage of the model and low at both the first stage and second stage of the model was (β = 0.171, 95% BC CI = [0.060, 0.294]), thus supporting H5.

## 5. Discussion

### 5.1. Theoretical Implications

This study has made theoretical contributions in several ways. First, it describes the concept of team-oriented citizenship pressure and reveals its positive impact on pro-group unethical behavior and the mediating role of moral disengagement, which enhances our understanding of the outcomes of citizenship pressure and antecedents of unethical pro-social behavior. While previous studies have confirmed the predictive effect of pressure on pro-social unethical behavior [8,43], citizenship pressure as a specific workplace pressure has not been discussed. Rather than the general path from pressure to pro-organizational unethical behavior, we demonstrate that citizenship pressure can also lead to pro-group unethical behavior by focusing on team-oriented citizenship pressure. Furthermore, previous research on the mechanism of citizenship pressure has often been viewed from the perspective of the conservation of resource theory [44]. By proposing moral disengagement as an explanatory mechanism for this new relationship, we extend the moral perspective of social cognitive theory to the field of citizenship pressure. Future research could explore the relationship between citizenship pressure and pro-social unethical behavior at both the organizational and team levels.

Second, by using self-serving political will as a moderator throughout the main effect, this study connects political will with moral disengagement and pro-social unethical behavior, responding to two academic calls. On the one hand, Moore, Detert, Trevino, Baker, and Mayer [15] called for the exploration of how individual differences adapt to self-regulation models of morality. The data results suggest that citizenship pressure no longer significantly predicts moral disengagement only when self-serving political will is low, indicating that self-serving political will is a sensitive condition for triggering moral disengagement under pressure. Employees with low self-serving political will have difficulty accepting the moral and emotional experiences of self-serving social impact behavior, and consider sacrificing the interests of others inappropriate [36]. Even in pressure environments that emphasize extra contributions, they adhere to higher moral standards rather than shirking moral responsibilities. On the other hand, Mo, Lupoli, Newman and Umphress [16] called for further research identifying the self-interested motivation of pro-social unethical behavior. The motives for pro-social unethical behavior at least partly involve benefiting others, but self-interest and the desire to harm others may also form the basis for such behavior [45]. We found that self-serving political will, as a self-serving motive, facilitates the evolution of moral disengagement into pro-group unethical behavior, providing new evidence that pro-social unethical behavior involves self-interested motivations.

### 5.2. Practical Implications

Under team-oriented citizenship pressure, team members with high self-serving political will bring both opportunities and challenges to the team and the organization. Managers can adjust relevant impact conditions according to the different management goals of construction or destruction. For team managers, subordinates with strong self-serving political will combined with team-oriented citizenship pressure can become a sharp edge for the team to use when competing against other teams. Rhee [46] found that collective pro-group unethical behavior positively predicted team performance, and that the effect was more evident when the leader was highly abusive. Therefore, if the team manager is a politically ambitious worker, it is possible to cultivate subordinates’ self-serving political will while creating a team-oriented citizenship pressure, enabling members to spontaneously attack other teams to gain an advantage. After all, in some cases, team competition is unavoidable (e.g., planned layoffs). Of course, if team managers consider a member’s pro-group unethical behavior to be unacceptable in terms of morality or long-term overall performance contributions, they should control the relevant factors.

For organization managers, the combination of self-serving political will and team-oriented citizenship pressure is a disadvantage. Pro-group unethical behavior means internal conflicts between the organization’s teams, and organizational-level managers should consider how to suppress pro-group unethical behavior. For example, regarding citizenship pressure, managers can monitor whether collective expectations that employees take on additional responsibilities are too high, and can promptly relieve them when necessary [1]. As for self-serving political will, political will is an acquired individual trait that can be shaped, and perceptions of superiors’ political behavior and individuals’ experience with the organization’s political history can predict employees’ political will [32]. Therefore, besides controlling through recruitment selection, managers should establish an open and explicit system for distributing benefits to control the level of organizational politics. As for moral disengagement, managers can reduce employees’ willingness and opportunities to shirk moral responsibilities through ethical training and redesigning job responsibilities [15].

### 5.3. Limitations and Future Research

The limitations of this study provide space for future exploration. Firstly, this study selectively applied self-serving political will, and subsequent research may consider the role of benevolent political will. In the literature review, we drew on established empirical evidence and discussed the reasons for only selecting self-serving political will as the boundary conditions for the model. It is worth noting that Blickle et al. [47] questioned the representativeness of benevolent political will for the altruistic political motives, and encouraged the development of new scales. Instead, the benevolent part is not driven by humanitarianism or altruism, but by personal interests associated with their specific group, and if the group succeeds, individuals’ self-interests will be promoted [48]. Through these different interpretations, we speculate that benevolent political will may be very suitable as a factor influencing pro-group unethical behavior. It is hoped that subsequent research can develop scales consistent with their understanding of benevolent political will, which can be applied to the relevant background of this study. As the two dimensions of political will may be a useful tool for identifying self-interest or altruistic motives for pro-social unethical behavior [16], this suggestion deserves full consideration.

Secondly, this study drew exploratory conclusions based on cross-sectional data, and its utility in inferring causality is limited. Although the impact pathway discussed should be irreversible in practical logic, and there is empirical evidence to support the causality between some factors, longitudinal or experimental research design is always necessary to supplement exploratory conclusions. Thirdly, pro-group unethical behavior is self-reported by employees, and future research can evaluate pro-group unethical behavior using other methods (such as leader evaluation and objective records) to see if our conclusions are still valid. Fourthly, individuals’ attitudes towards OCBs and pro-social unethical behavior are influenced by individualism–collectivism [16,49], and future research could be conducted in countries and regions with more individualistic cultures to verify whether the conclusions have cross-cultural generalizability. Finally, pro-group unethical behavior reflects organizational infighting, and is a phenomenon that is harmful to socio-economic entities. However, there is limited research discussing it. Future organizational research may pay more attention to this behavior. In addition to micro-psychological insights such as those in this study, a macro perspective, through laws and social factors related to human resources [50,51], may also enhance our understanding of and interventions in such behavior.

## 6. Conclusions

This study depicts the phenomenon of employees implementing pro-group unethical behavior under team-oriented citizenship pressure, which presents opportunities or challenges to the team- and organizational-level managers. Drawing on moral discussions of social cognitive theory, this study proposes a dual-stage moderated mediation model. The results confirm that citizenship pressure positively predicts pro-group unethical behavior through moral disengagement. Self-serving political will is a positive moderator in the first stage, the second stage, and the mediating effect. The findings herein may contribute to enhancing our theoretical understanding and practical management of citizenship pressure, pro-group unethical behavior, and self-serving political will.

## Figures and Tables

**Table 1:** Comparison of measurement models.

Models	Factors	χ^2^	df	χ^2^/df	RMSEA	CFI	IFI	TLI
4-factor model	CP, SPW, MD, PUB	849.895	269	3.159	0.064	0.956	0.956	0.951
3-factor model a	CP + SPW, MD, PUB	3014.750	272	11.084	0.138	0.792	0.793	0.771
3-factor model b	CP, SPW, MD + PUB	2379.284	272	8.747	0.121	0.840	0.841	0.824
2-factor model	CP + SPW, MD + PUB	4543.783	274	16.583	0.172	0.677	0.677	0.646
1-factor model	CP + SPW + MD + PUB	6468.757	275	23.523	0.207	0.531	0.532	0.488

Notes: N = 527. CP, citizenship pressure; SPW, self-serving political will; MD, moral disengagement; PUB, pro-group unethical behavior.

**Table 2:** Means, standard deviations, correlations, and reliabilities of variables.

Variables	M	SD	1	2	3	4	5	6	7	8
1. Gender	1.620	0.485								
2. Age	30.590	7.031	−0.113 **							
3. Education	2.620	0.801	−0.021	−0.253 ***						
4. Tenure	6.690	6.152	−0.005	0.768 ***	−0.190 ***					
5. CP	3.328	0.912	0.037	0.075	0.051	0.052	(0.915)			
6. SPW	2.832	1.283	0.047	0.074	−0.117 **	0.141 **	0.344 ***	(0.958)		
7. MD	1.845	1.024	−0.070	0.074	0.009	0.061	0.304 ***	0.415 ***	(0.958)	
8. PUB	1.775	1.095	−0.048	0.062	−0.015	0.040	0.306 ***	0.402 ***	0.730 ***	(0.964)

Notes: N = 527. ** *p*< 0.01. *** *p*< 0.001. CP, citizenship pressure; SPW, self-serving political will; MD, moral disengagement; PUB, pro-group unethical behavior.

**Table 3:** Regression outcomes.

	Model 4		Model 58	
	MD	PUB	MD	PUB
Control variables				
Gender	−0.167 (0.089)	−0.002 (0.068)	−0.176 * (0.083)	−0.044 (0.068)
Age	0.003 (0.010)	0.002 (0.007)	0.010 (0.009)	0.006 (0.007)
Education	0.004 (0.055)	−0.037 (0.042)	0.060 (0.052)	−0.004 (0.042)
Tenure	0.005 (0.011)	−0.004 (0.008)	−0.006 (0.010)	−0.009 (0.008)
Independent variable				
CP	0.341 *** (0.047)	0.113 ** (0.038)	0.185 *** (0.047)	0.076 * (0.038)
Mediator				
MD		0.751 *** (0.033)		0.567 *** (0.053)
Moderator				
SPW			0.282 *** (0.034)	0.132 *** (0.031)
CP × SPW			0.078 * (0.032)	
MD × SPW				0.121 *** (0.033)
R^2^ (MSE)	0.101 (0.951)	0.542(0.555)	0.224 (0.825)	0.561 (0.534)
	**Effect**	**95% BC CI**	**Effect**	**95% BC CI**
Total	0.369 *** (0.050)	[0.270, 0.468]		
Direct	0.113 ** (0.038)	[0.039, 0.187]	0.076 * (0.038)	[0.001, 0.151]
Indirect	0.256 (0.044)	[0.175, 0.346]	0.105 (0.026)	[0.057, 0.160]
Indirect (SPW high-low)			0.171 (0.060)	[0.060, 0.294]

Notes: N = 527. Unstandardized coefficients are reported with standard errors in parentheses. Bootstrap sample size = 5000. * *p*< 0.05. ** *p*< 0.01. *** *p*< 0.001. CP, citizenship pressure; SPW, self-serving political will; MD, moral disengagement; PUB, pro-group unethical behavior.

## Data Availability

The data that support the findings of this study are available from the corresponding author upon reasonable request.
